# Pharmaceutical Disorganisation

**Published:** 1911-12-09

**Authors:** 


### PHARMACEUTICAL DISORGANISATION.

### Its Causes and a Suggested Remedy.

BY A PROVINCIAL HOSPITAL PHARMACIST.

The pharmaceutical craft was until recently one of the most disorganised bodies of the community in the United Kingdom. The fact is very remarkable when one considers that the State has on two occasions found it necessary to pass Acts of Parliament regulating the practice of pharmacy and the handling of poisonous substances to those persons who had, by the examinations required by and supervised by his Majesty's Privy Council, proved their right to be placed on the Register of Chemists and Druggists or Pharmacists and Pharmaceutical Chemists. Naturally one would have thought that if the State, by its Houses of Parliament, found it necessary to bring out these regulations, the pharmacists would also have found it expedient to form themselves into one strong association which could, effectively, have looked after and protected the interests of the profesion. But for some reason, not easily understood by the ordinary pharmacist of to-day, the organisation of pharmacy was evidently not fully recognised as being of great importance. A well-known member of the medical profession recently asked how it was that a person, not being a member of the Pharmaceutical Society of Great Britain, claimed to be a registered pharmacist, and he seemed to be considerably astonished to find that this could be the case and that the membership of the chief pharmaceutical organisation was voluntary, although restricted to those persons whose names were on the Register.

It is well known in pharmacy circles that every person who passes the qualifying examination is given a notice stating he or she is now eligible to claim membership, and it is to be regretted that a large number does not take this important step.

### Local Associations.

So far we have practically laid all the blame for this state of affairs on the individual, but it is only fair to say that many of the most loyal and energetic members of the Pharmaceutical Society believe the organisation of the Society is greatly to blame for the present disorganised state of the profession. As the practice of pharmacy is of necessity spread throughout the kingdom, it is unnecessary to say every man cannot be in direct touch with the London body, but it is possible for every registered person to be a member of the local associations which have been established in the provinces. The organisation of these bodies is peculiar, inasmuch as they work under different constitutions or rules. This peculiarity is very striking and remarkable when we find that three distinct classes exist: (1) The associations who admit to membership those persons only who are on the Register; (2) Persons on the Register who are in business (employers); this class prohibits a great many men who hold the same qualification. (3) The third series admit all persons who devote their whole time to pharmacy. It is difficult to find the causes which influenced these different regulations.

No doubt the first class of these local associations is the most suitable for the pharmaceutical profession, and without doubt the most capable of working for the good of the craft. The second class seems to have been founded under the old system of organisation, when the pharmacist who was in business on his own account looked upon his qualified assistants only as servants and not as fellow-members of the profession. The Pharmaceutical Society held to this view for many years, giving three classes in their register of members: (*a*) Pharmaceutical chemists, (*b*) chemists and druggists *in business*, (*c*) chemists and druggists *not* in business. This distinctive classification was keenly resented, and it is quite evident this feeling had a great deal to do with the present disorganisation. The third class, although open to grave criticism, is perhaps the beginning of that friendly feeling which is gradually becoming stronger amongst the craft.

A few months ago the Council of the Pharmaceutical Society decided to appoint a special committee to deal with local associations. This was probably due to the definite expressions of opinion which were given in the columns of the pharmaceutical press, and may also have been done because of the appeals of the local bodies for official information. Previously there was no department to deal with these matters, therefore every provincial society was working independently of the official council and of each other, and so much, if not all, the work done in the provinces was practically useless as far as the craft in general was concerned.

### Divisional Secretaries.

These officers were appointed to act as representatives of the Society in all the Parliamentary divisions of the country, but this system has not been so successful as anticipated. In the first place, these officials have no connection in their official capacity with the local associations, and in several instances are not on the local councils. This must prevent good work being done, as all know that important points are raised and discussed at such meetings which cannot be dealt with at the general meetings. Recently the Pharmaceutical Society has handed over to the local associations the privilege of electing or rather suggesting the names of men to act as secretaries. This is a step in the right direction, and now gives the local societies the opportunity of adding these men to their councils by virtue of their office. But it is remarkable that the Society, after appointing these gentlemen, does not take them into its confidence. The system is to blame for the failure and not the men, but perhaps under the new organising committee a new start will be made.

